# Discovery and molecular insights of a novel pea-derived antioxidant peptide: From quantum chemical insights to oxidative stability of walnut oil

**DOI:** 10.1016/j.fochx.2026.104474

**Published:** 2026-09-18

**Authors:** Jingna Peng, Tingmei Qi, Qi Lin, Xinyong Zhang, Yue Wang, Yuanyuan Bao

**Affiliations:** a College of Food Science and Technology, Yunnan Agricultural University, Kunming 650201, Yunnan, China; b College of Agronomy and Biotechnology, Yunnan Agricultural University, Kunming 650201, Yunnan, China

**Keywords:** Defatted pea protein, Antioxidant peptides, Quantum chemical calculation, Molecular docking, Molecular dynamics simulation, Lipid oxidation

## Abstract

This study identified novel antioxidant peptides from defeasated pea protein and characterized their molecular properties and protective effects in a lipid system. Following ultrafiltration, Sephadex G-15 purification, Nano LC–MS/MS identification, and in silico screening, five unreported peptides (F1–F5) were selected for evaluation. Antioxidant activities varied across DPPH, ABTS, and hydroxyl radical assays, reflecting sequence- and assay-specific differences. Integrated experimental and computational analyses identified GIPYWTY (F1) as the lead peptide. Molecular docking revealed favorable F1–Keap1 interactions, while quantum-chemical analyses highlighted radical-reactive regions. A 100 ns molecular dynamics simulation confirmed complex stability, with an MM/PBSA binding free energy of −36.73 kJ/mol. In walnut oil, 0.075% (*w*/w) F1 supplementation attenuated increases in peroxide and acid values and partially preserved antioxidant capacity during 10 d of accelerated oxidation at 60 °C. These findings establish GIPYWTY as a promising pea-derived antioxidant peptide for mitigating oxidative deterioration in lipid-rich foods.

## Highlights

• Five previously unreported antioxidant peptides were identified from pea protein.
• GIPYWTY was selected as the lead peptide by integrated evaluation.
• GIPYWTY showed favorable and stable interaction with Keap1.
• Quantum chemistry revealed potential radical-reactive sites in the peptides.
• GIPYWTY retarded walnut oil oxidation under accelerated conditions.

## Graphical abstract

**Figure:**
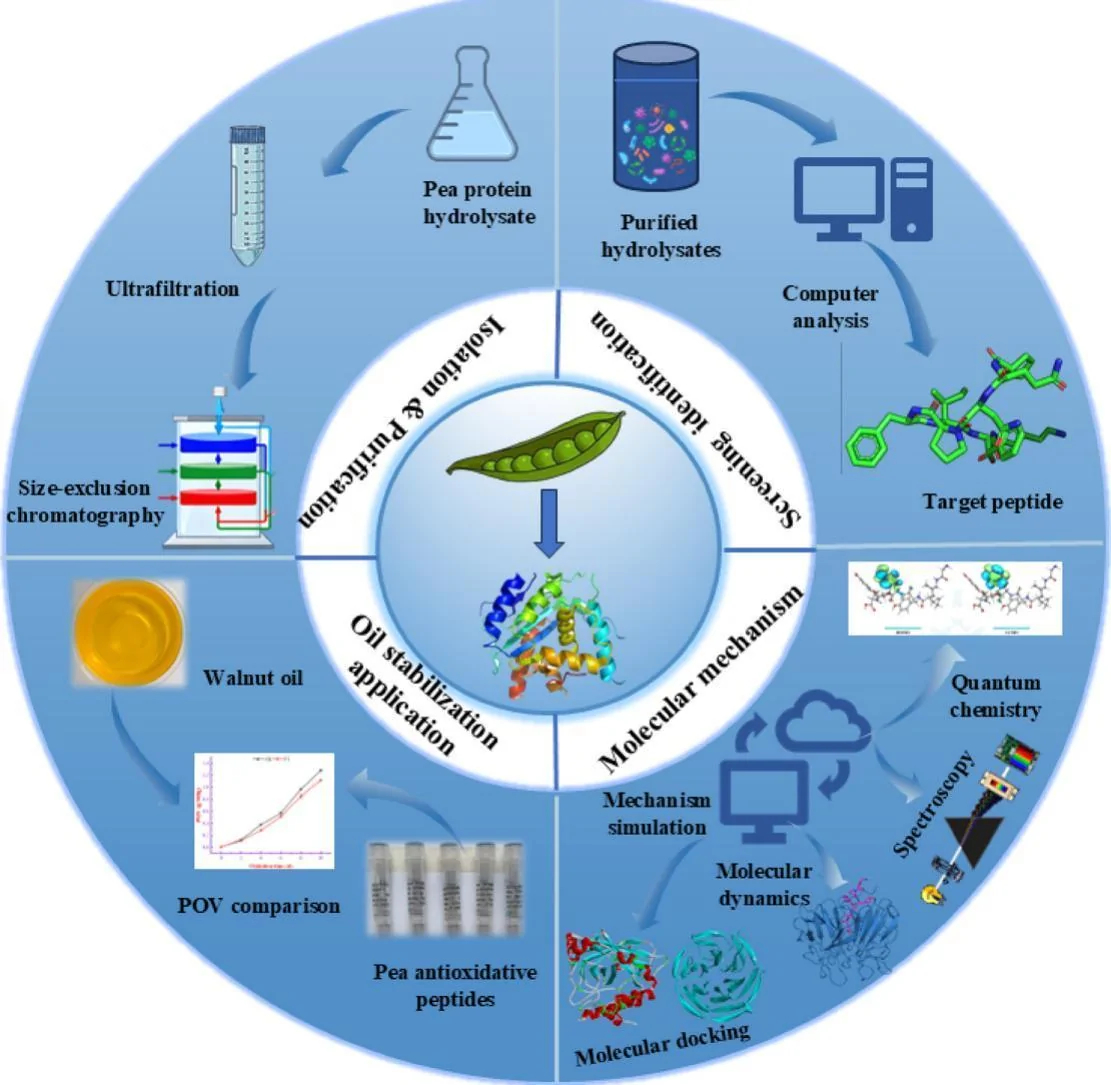

## 1 Introduction

In recent years, food-derived antioxidant peptides have attracted increasing attention as potential natural alternatives to synthetic antioxidants because of their radical-scavenging capacity and potential applications in food systems. A growing number of antioxidant peptides have been identified from edible plant proteins and plant-derived agro-food processing by-products (Wong et al., 2020). For example, walnut-derived peptides have exhibited both extracellular and cellular antioxidant activities and protected cells against oxidative damage (Wang et al., 2022). Similarly, enzymatic hydrolysis of brewers' spent grain proteins produced peptide fractions with strong DPPH and superoxide radical scavenging activities, demonstrating the potential of this abundant processing by-product as a source of natural antioxidant peptides (Abeynayake et al., 2022).

Beyond chemical and cellular antioxidant assays, increasing attention has been directed toward evaluating antioxidant peptides in food-related systems. Plant-derived antioxidant peptides have been reported to inhibit oxidative reactions through multiple mechanisms, supporting their potential application in food preservation (Zhu et al., 2024). More direct evidence has also been obtained in lipid-containing food systems. Antioxidant peptide fractions enriched from zinc ion-induced oat polypeptides, together with their representative peptide components, exhibited inhibitory effects on lipid oxidation as well as tyrosinase activity (Yin et al., 2025). In addition, the Tartary buckwheat-derived antioxidant peptide WPWR was incorporated into a peptide–polysaccharide composite film, which delayed quality deterioration and extended the storage life of strawberries (Zhang et al., 2025). Collectively, these findings demonstrate the potential of plant-derived antioxidant peptides as natural functional ingredients for controlling oxidative deterioration in foods and provide a rationale for further investigating their structure–activity relationships and performance in real food matrices.

The Keap1/Nrf2 signaling pathway plays a central role in the cellular defense against oxidative stress, and Keap1 has consequently emerged as an important molecular target for investigating the antioxidant mechanisms of food-derived peptides. Molecular docking provides a useful computational approach for evaluating peptide–Keap1 molecular recognition and identifying amino acid residues that may contribute to peptide binding. For example, molecular docking and molecular dynamics simulations demonstrated that soybean-derived antioxidant peptides could interact with key regions of Keap1, providing a molecular basis for their potential involvement in Keap1-mediated antioxidant regulation (Zhang, Yan, et al., 2024). Similarly, Igbokwe et al. screened coix seed-derived antioxidant peptides by molecular docking against the Keap1-Kelch domain and subsequently identified FFDR as a promising peptide capable of modulating antioxidant-related responses in HepG2 cells (Igbokwe et al., 2024). Molecular docking can also be applied to characterize direct interactions between antioxidant peptides and radical species. Fan et al. identified four novel duck liver-derived antioxidant peptides and showed that hydrogen-bonding interactions contributed substantially to their association with free radical molecules (Fan et al., 2023). In summary, these studies demonstrate that molecular docking can provide valuable molecular-level information on both peptide–protein recognition and peptide–radical interactions.

Quantum chemical calculations provide complementary information by characterizing the electronic structure, electron-donating propensity, and potential reactive sites of antioxidant peptides. Frontier molecular orbital descriptors, including the highest occupied molecular orbital (HOMO), lowest unoccupied molecular orbital (LUMO), and HOMO–LUMO energy gap, together with atomic charge and Fukui function analyses, have increasingly been used to investigate peptide structure–activity relationships. Li et al. (2025) combined deep learning with quantum chemical calculations to identify antioxidant peptides and demonstrated that differences in HOMO–LUMO energy gaps and reactive atomic sites were associated with their antioxidant potential. Likewise, molecular docking combined with quantum chemical calculations was used to screen antioxidant peptides from Tartary buckwheat, leading to the identification of WPWR, FLQL, and HGLFSPF as peptides with high predicted bioactivity (Zhang et al., 2025). Therefore, integrating molecular docking with quantum chemical calculations provides a complementary strategy for linking molecular interactions and electronic reactivity to the antioxidant activity of food-derived peptides.

Pea (*Pisum sativum* L.) is an important legume crop with broad adaptability and considerable nutritional and economic value. Its seeds are rich in protein, starch, dietary fiber, and other nutrients, making pea an increasingly important source of plant-based protein. Pea protein possesses a favorable amino acid profile, particularly with respect to lysine, although sulfur-containing amino acids are relatively limited (Wang et al., 2022). However, the relatively low solubility and limited techno-functional properties of native pea protein can restrict its broader application in food systems (Shanthakumar et al., 2022). Accordingly, various modification strategies have been investigated to improve its structural and functional properties, including high-intensity ultrasound combined with pH shifting and interactions with polyphenols (Kim et al., 2025; Zhang, J., et al., 2022). Enzymatic hydrolysis provides an additional strategy for converting pea proteins into bioactive peptide fractions, and pea protein hydrolysates have demonstrated antioxidant and other biological activities (Ndiaye et al., 2012). Nevertheless, systematic understanding of the relationships among peptide sequence, molecular structure, electronic reactivity, antioxidant mechanisms, and performance in lipid food systems remains limited for pea-derived antioxidant peptides.

In this study, antioxidant peptides were prepared from pea protein hydrolysates and fractionated by ultrafiltration and Sephadex G-15 gel filtration chromatography, followed by peptide identification using Nano LC-MS/MS and in vitro antioxidant activity screening. The three-dimensional structures of selected peptides were predicted using AlphaFold 3, and molecular docking was employed to characterize their interactions with Keap1 radical species. Quantum chemical calculations, including frontier molecular orbital, atomic charge, and Fukui function analyses, were further performed to identify potential electron- and hydrogen-donating reactive sites and to elucidate peptide structure–activity relationships. Spectroscopic characterization and molecular dynamics simulations were integrated to provide complementary information on peptide conformation and molecular interactions. Finally, the antioxidant performance of the selected peptide GIPYWTY was evaluated in walnut oil under accelerated oxidation conditions. By integrating peptide identification, experimental antioxidant evaluation, molecular docking, quantum chemical calculations, structural characterization, molecular dynamics simulations, and food-matrix validation, this study aimed to elucidate the molecular basis of pea-derived antioxidant peptides and assess their potential as natural antioxidants for lipid-containing food systems.

## 2 Materials and methods

### 2.1 Materials and reagents

Pea flour was obtained from Yunnan Yulongsheng Agricultural Science and Technology Co., Ltd. (Yuxi, Yunnan, China). The pea flour was defatted with hexane at a flour-to-solvent ratio of 1:5. After defatting, the samples were air-dried to remove residual hexane. Pea protein was subsequently extracted from the defatted flour using an alkaline solubilization–acid precipitation method according to previous studies (Luo et al., 2022). The resulting protein precipitate was collected by centrifugation (4000 rpm, 10 min), washed, and freeze-dried. The obtained defatted pea protein was stored at 4 °C until further use.

Six antioxidant peptide candidates, GIPYWTY (F1), APGPIWTP (F2), GVAPGPIWTP (F3), APGPVWTP (F4), TPPGIPYW (F5), and PQNPFIFK (F6), were chemically synthesized at a purity of 98% by Synpeptide Jietai Biochemical Co., Ltd. (Nanjing, China).

### 2.2 Preparation and fractionation of defatted pea protein hydrolysates

Defatted pea protein was dispersed in deionized water at a ratio of 1:50 (g/mL) and heated at 90 °C for 10 min. Hydrolysis was performed at pH 7.0 for 3 h using a mixed protease system of alkaline protease and flavourzyme (3:1) at a total enzyme dosage of 10,000 U/g protein. The enzymes were then inactivated in boiling water for 15 min, followed by centrifugation at 6000 rpm for 10 min at 4 °C.

The supernatant was sequentially fractionated using 3 and 1 kDa ultrafiltration devices at 5000 ×g and 4 °C for 1 h to obtain >3 kDa, 1–3 kDa, and < 1 kDa fractions. The <1 kDa fraction, which showed the highest antioxidant activity, was further purified using a Sephadex G-15 column (16 mm × 60 cm). A 2 mL sample (40 mg/mL) was eluted with deionized water at 1 mL/min, and fractions were collected every 2.4 min and monitored at 280 nm. Three major fractions (P1–P3) were obtained, and P2, which exhibited the highest antioxidant activity, was selected for Nano LC–MS/MS analysis.

### 2.3 Peptide sequence identification and in silico screening

The P2 fraction was analyzed by Nano LC–MS/MS using a nanoElute2 nano-UPLC system coupled to a timsTOF Pro2 mass spectrometer with a nano-electrospray ionization source (Bruker, Germany). Approximately 200 ng of peptides were separated on a PePSep C18 column (1.9 μm, 75 μm × 15 cm) using water containing 0.1% formic acid (A) and acetonitrile containing 0.1% formic acid (B) over a 45-min gradient. Data were acquired in DDA-PASEF mode over an MS1 range of *m/z* 100–1700. Collision energy increased linearly from 20 eV at 1/K0 = 0.6 *V*·s/cm^2^ to 59 eV at 1/K0 = 1.6 *V*·s/cm^2^.

Raw data were processed using SpectroMine (version 4.2.230428.52329) with the Pulsar search engine against a *Pisum sativum* UniProt FASTA database. Searches were performed with No-Enzyme specificity, carbamidomethylation of C as a fixed modification, and oxidation of M and protein N-terminal acetylation as variable modifications. Precursor and fragment mass tolerances were set to 20 ppm, and peptide-spectrum matches and peptides were filtered at FDR ≤0.01.

Identified peptides were further screened using PeptideRanker (>0.5), AllergenFP v1.0, ToxinPred, ProtParam, PepDraw, and BIOPEP-UWM. Six previously unreported candidate sequences (F1–F6) were initially identified. F6 showed comparatively lower predicted radical-scavenging activity and was excluded, whereas F1–F5 were retained for subsequent experimental evaluation.

### 2.4 Determination of in vitro antioxidant activity

#### 2.4.1 DPPH, ABTS, and hydroxyl radical scavenging activities

Following the methods described in previous studies, we determined the DPPH, ABTS, and hydroxyl radical scavenging activities of the peptide samples with some minor modifications (Lin et al., 2024). Absorbance was measured at 517, 734, and 510 nm for the DPPH, ABTS, and hydroxyl radical assays, respectively. All measurements were performed in triplicate, and radical scavenging activities were expressed as percentages.

#### 2.4.2 Reducing power

The reducing power of the peptide samples was determined using a slightly modified method adapted from previous work (Liu, C., et al., 2018). Absorbance was measured at 700 nm, with higher absorbance indicating greater reducing power. All measurements were performed in triplicate.

### 2.5 Computational analyses

#### 2.5.1 Three-dimensional structure prediction

The three-dimensional structures of the selected peptides were predicted using AlphaFold 3. Prediction confidence was evaluated using the predicted local distance difference test (pLDDT) score, with values >90 indicating very high local confidence in the predicted structure.

#### 2.5.2 Molecular docking

Peptide structures were constructed using ChemDraw 22.0 and subsequently subjected to energy minimization using ChemBio3D. The optimized structures were saved in PDB format and prepared using AutoDockTools by adding hydrogen atoms and assigning charges. The three-dimensional structure of Keap1 (PDB ID: 4IFJ) was obtained from the RCSB Protein Data Bank. Crystallographic water molecules and the co-crystallized ligand were removed using PyMOL prior to docking. Molecular docking was performed using AutoDock Vina, with Keap1 defined as the receptor and the peptides as ligands. The docking grid was centered at *x* = 2.320, *y* = 31.428, and *z* = 68.100 Å, with dimensions of 46.88 × 46.88 × 46.88 Å. Nine docking poses were generated, and the pose with the most favorable binding energy was selected for subsequent interaction analysis and visualization using PyMOL (Lee et al., 2024).

#### 2.5.3 Quantum chemical calculations

Quantum chemical calculations for peptides F1–F5 were performed using Gaussian 16 (Revision C.01). Geometry optimization was conducted at the B3LYP-D3(BJ)/6-31G(d,p) level with ethanol represented using the polarizable continuum model (PCM). Single-point energy calculations were subsequently performed at the B3LYP-D3(BJ)/6-311G(d,p) level using the SMD implicit solvation model for ethanol. Frontier molecular orbitals (HOMO and LUMO), atomic charge distributions, and Fukui functions were analyzed using Multiwfn, and molecular orbitals and reactive sites were visualized using VMD.

#### 2.5.4 Molecular dynamics simulations

Molecular dynamics (MD) simulations of the F1–Keap1 complex were performed using GROMACS 2023.3. The CHARMM36 force field was applied to both Keap1 and F1, and the complex was solvated using the TIP3P water model. Following steepest-descent energy minimization, the system was equilibrated for 100 ps under the NVT ensemble and subsequently for 100 ps under the NPT ensemble. The temperature and pressure were maintained at 313.15 K and 1 bar using the *V*-rescale thermostat and C-rescale barostat, respectively. Bonds involving hydrogen atoms were constrained using the LINCS algorithm. A cutoff of 1.2 nm was applied to van der Waals interactions, and long-range electrostatic interactions were treated using the particle-mesh Ewald (PME) method. A 100 ns production simulation was then performed with a time step of 2 fs. Root-mean-square deviation (RMSD), root-mean-square fluctuation (RMSF), radius of gyration (Rg), solvent-accessible surface area (SASA), and intermolecular hydrogen bonds were analyzed. Binding energetics were estimated using the MM/PBSA method. Trajectories and representative structures were visualized using VMD 1.9.3 and PyMOL 3.1.

### 2.6 Spectroscopic and morphological characterization

#### 2.6.1 UV–visible spectroscopy

Defatted pea protein and peptide samples were dissolved in 0.1% NaOH and phosphate-buffered saline (PBS), respectively. UV–visible spectra were recorded over the wavelength range of 230–400 nm at a scanning rate of 10 nm/s.

#### 2.6.2 Intrinsic fluorescence spectroscopy

Defatted pea protein and peptide samples were dissolved in 0.1% NaOH and PBS, respectively. Intrinsic fluorescence spectra were recorded at an excitation wavelength of 280 nm over an emission wavelength range of **300–500 nm.**

#### 2.6.3 **Circular dichroism spectroscopy**

The method for determining Circular dichroism (CD) spectra was based on previous reports, with minor modifications (Li, S., et al., 2023). Samples were placed in a 1 mm quartz cuvette and scanned over 190–400 nm. The far-UV region (200–260 nm) was used for secondary-structure analysis using the BeStSel method (Micsonai et al., 2022).

#### 2.6.4 **Fourier transform infrared spectroscopy**

Freeze-dried samples were thoroughly mixed with anhydrous KBr and compressed into transparent pellets. Following established protocols, we recorded FTIR spectra in the wavenumber range of 4000–400 cm^−1^ with 64 scans, with slight modifications (Jia et al., 2024).

#### 2.6.5 **Scanning electron microscopy**

Freeze-dried samples were mounted on conductive adhesive, sputter-coated with gold under vacuum, and examined using scanning electron microscopy at different magnifications.

### 2.7 Oxidative stability of walnut oil supplemented with F1

F1 was added to walnut oil at 0.075% (*w*/w), while untreated walnut oil served as the control (CK). Each sample contained 35 g of walnut oil and was sonicated in an ice-water bath for 30 min to uniformly disperse the peptide. Accelerated oxidation was conducted using the Schaal oven test at 60 ± 1 °C for 10 d. Independent samples were prepared for each sampling time (0, 2, 4, 6, 8, and 10 d), and the oil samples were gently mixed every 4–6 h during incubation. Peroxide value (POV), acid value (AV), malondialdehyde (MDA) content, total phenolic content, and DPPH, ABTS, and hydroxyl radical-scavenging activities were determined to evaluate oxidative deterioration and antioxidant capacity.

### 2.8 **Statistical analysis**

All experiments were performed independently in triplicate, and data are expressed as mean ± standard deviation (SD). Statistical analyses were performed using SPSS software. One-way analysis of variance (ANOVA) followed by Tukey's multiple-comparison test was used to compare antioxidant activities among peptide samples. For the walnut oil experiment, the effects of treatment (CK and F1), oxidation time, and their interaction were evaluated using two-way ANOVA. Differences were considered statistically significant at *P* < 0.05. Origin was used for graphical presentation.

## 3 Results and discussion

### 3.1 Analysis of antioxidant activity of ultrafiltered fractions of defatted pea protein

Three peptide fractions with molecular weights of >3 kDa, 1–3 kDa, and < 1 kDa were obtained by ultrafiltration, and their DPPH, ABTS, and hydroxyl radical-scavenging activities and reducing power were evaluated at different concentrations (Fig. 1A–D). All three fractions exhibited concentration-dependent antioxidant activities, although their responses varied among the different assays. The <1 kDa fraction showed significantly higher DPPH and hydroxyl radical-scavenging activities than the >3 kDa and 1–3 kDa fractions over the tested concentration range (*P* < 0.05). For ABTS radical scavenging, the <1 kDa fraction generally exhibited the highest activity at 1.8–3.0 mg/mL, whereas the >3 kDa fraction showed the highest activity at 1.2 mg/mL. Similarly, the reducing power of the <1 kDa fraction was significantly higher than that of the higher-molecular-weight fractions at 1.8–3.0 mg/mL (*P* < 0.05). Overall, the <1 kDa fraction exhibited the strongest combined antioxidant performance and was therefore selected for subsequent purification.

**Fig. 1:**
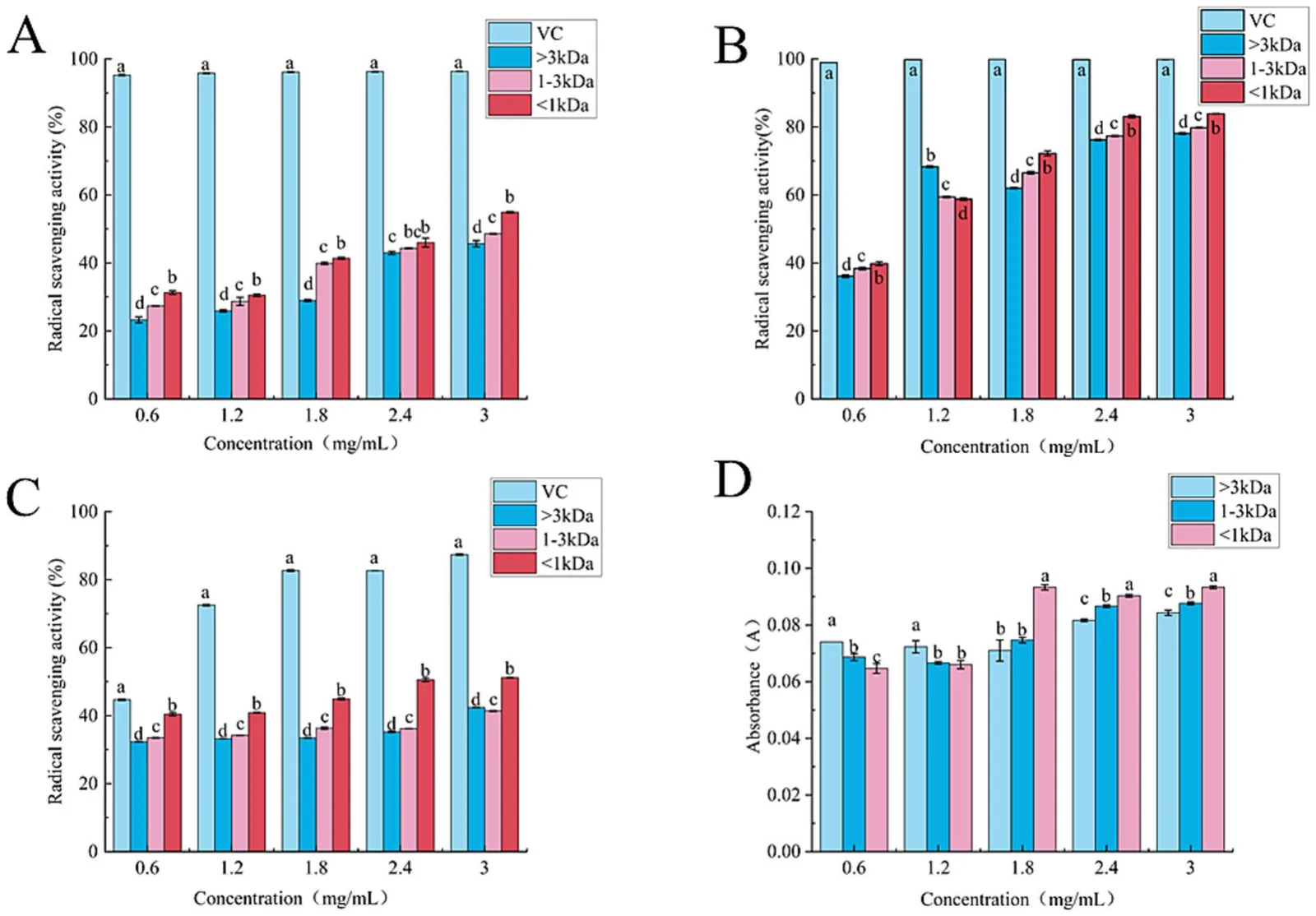
Antioxidant activities of different molecular-weight fractions obtained from defatted pea protein hydrolysate. (A) DPPH radical-scavenging activity; (B) ABTS radical-scavenging activity; (C) hydroxyl radical-scavenging activity; and (D) reducing power. VC, vitamin C positive control, was included only in panels A–C. Values are expressed as mean ± SD (*n* = 3). Different lowercase letters indicate significant differences among groups at the same concentration (*P <* 0.05), as determined by one-way ANOVA followed by Tukey's test.

The enhanced antioxidant activity of low-molecular-weight peptides may be associated with reduced steric hindrance and greater accessibility of radical-reactive residues, although peptide sequence and amino acid composition also play important roles in determining antioxidant capacity (Zhang et al., 2026). Similar molecular-weight-dependent antioxidant behavior has been reported for selenium-enriched tea peptides, in which low-molecular-weight fractions exhibited relatively strong radical-scavenging activity (Wei et al., 2023).

### 3.2 Identification and screening of antioxidant peptide candidates

Nano LC–MS/MS analysis of the <1 kDa fraction identified 726 peptide sequences using SpectroMine with the Pulsar search engine. Based on predicted bioactivity, allergenicity, toxicity, physicochemical properties, and antioxidant potential, eight peptide candidates were initially selected for further screening. Sequence comparison using the BIOPEP-UWM database indicated that two peptides had been previously reported, whereas six peptide sequences were considered previously unreported and designated F1–F6 (Fig. 2 and Table 1). Low-molecular-weight peptides are considered promising antioxidant candidates because their small size may facilitate the accessibility of reactive residues and interactions with radical species (Zhao et al., 2023).

**Fig. 2:**
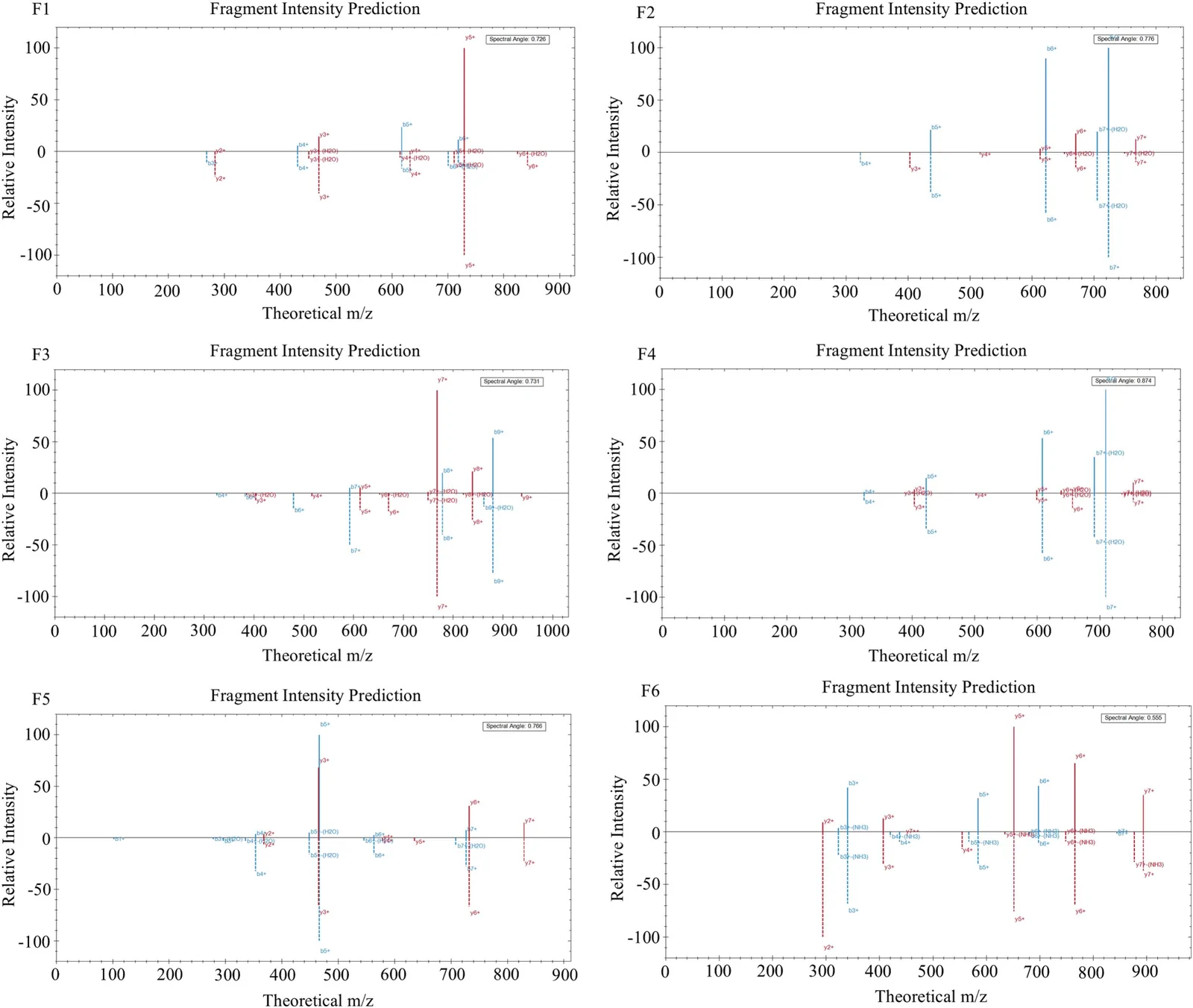
Experimental and theoretical MS/MS fragmentation spectra of six peptide candidates identified from the <1 kDa fraction of defatted pea protein hydrolysate. (A) F1, GIPYWTY; (B) F2, APGPIWTP; (C) F3, GVAPGPIWTP; (D) F4, APGPVWTP; (E) F5, TPPGIPYW; and (F) F6, PQNPFIFK. Matched fragment ions and spectral-angle values are indicated.

**Table 1:** In silico physicochemical and antioxidant-related properties of six pea-derived peptide candidates identified from the <1 kDa fraction.

Peptide	F1	F2	F3	F4	F5	F6
Sequence	*GIPYWTY*	*APGPIWTP*	*GVAPGPIWTP*	*APGPVWTP*	*TPPGIPYW*	*PQNPFIFK*
Molecular weight (Da)	899.00	837.96	994.14	823.93	930.06	990.15
PeptideRanker score	0.83	0.90	0.79	0.84	0.85	0.87
Allergenicity	No	No	No	No	No	No
Toxicity	No	No	No	No	No	No
Instability index	−43.96	14.04	13.23	38.11	5.65	34.51
Stability	Yes	Yes	Yes	Yes	Yes	Yes
Hydrophobicity score	4.81	7.01	7.7	7.67	5.8	8.06
Hydrophobic amino acid proportion	0.43	0.75	0.7	0.75	0.63	0.38
Predicted radical-scavenging activity	0.58	0.49	0.51	0.49	0.52	0.41
Predicted metal-chelationscore	0.20	0.21	0.22	0.21	0.24	0.25

***Note:*** F1–F6 were identified as previously unreported peptide candidates based on BIOPEP-UWM database screening. F1–F5 were selected for experimental validation, whereas F6 was excluded due to its lower predicted radical-scavenging score.

The antioxidant potential of peptides is closely associated with their amino acid composition, sequence arrangement, and exposure of reactive residues. Hydrophobic and aromatic amino acids, including Tyr, Trp, Phe, Pro, Ile, and Val, have been reported to contribute to radical scavenging through hydrogen donation, electron transfer, and hydrophobic interactions (Matsui et al., 2018; Shi et al., 2022). As shown in Table 1, all six candidate peptides were predicted to be non-allergenic, non-toxic, and stable, with PeptideRanker scores above 0.5. The hydrophobic amino acid proportions of F1–F6 ranged from 0.38 to 0.75, indicating differences in their potential interactions with free radicals and lipid environments.

Among the six candidates, F1–F5 exhibited relatively higher predicted radical-scavenging scores (0.49–0.58), whereas F6 showed the lowest score (0.41) accompanied by the lowest hydrophobic amino acid proportion (0.38). In addition, F1–F5 contained aromatic or hydrophobic residues, particularly Trp and Tyr, which may contribute to radical stabilization. Therefore, F6 was excluded, and F1–F5 were retained for subsequent experimental evaluation. The predicted physicochemical characteristics and antioxidant-related properties provided a basis for further investigation of the structural features and antioxidant mechanisms of pea-derived peptides.

### 3.3 Predictive analysis of the three-dimensional structures of pea antioxidant peptides

The three-dimensional structures of F1–F5 were predicted using AlphaFold 3 (Fig. 3). AlphaFold 3 provides an advanced deep-learning-based approach for biomolecular structure prediction using a diffusion-based architecture (Abramson et al., 2024). The predicted peptide structures were mainly composed of regions with high or very high pLDDT scores, indicating reliable local confidence for most residues. As shown in Fig. 3, most regions of F1–F5 exhibited pLDDT values >90, whereas limited regions, particularly in F4 and F5, showed relatively lower confidence. These predicted structures were subsequently used as initial models for molecular docking and other computational analyses. Nevertheless, pLDDT represents prediction confidence rather than direct experimental validation of peptide conformations.

**Fig. 3:**
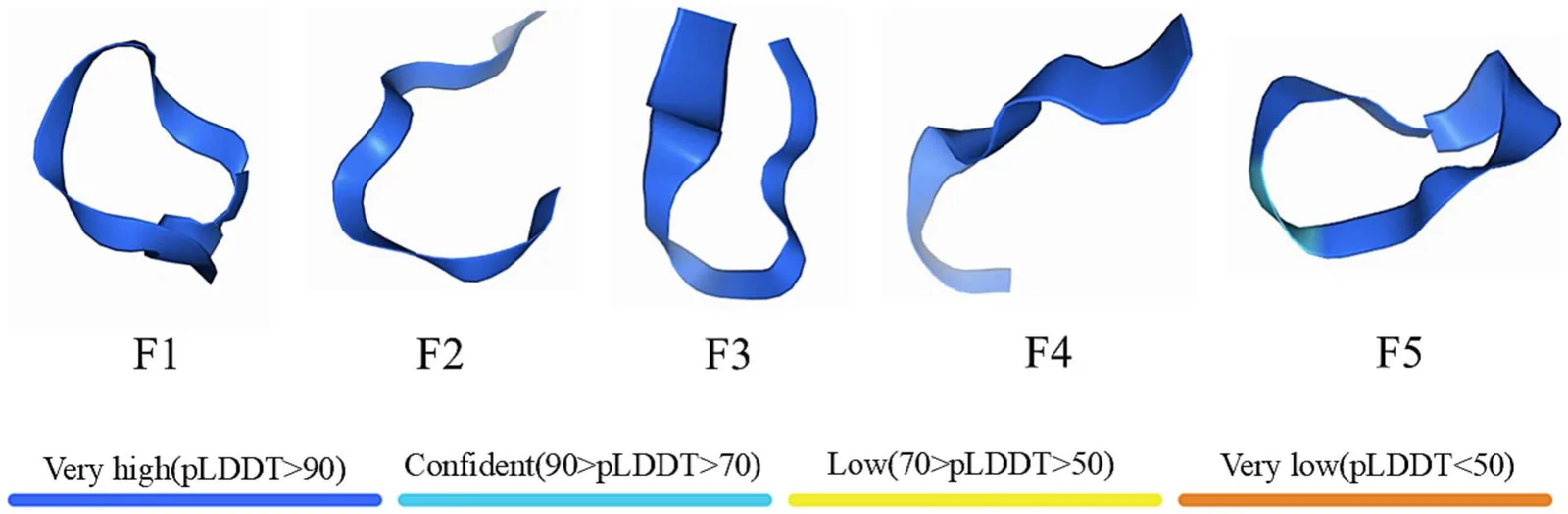
AlphaFold 3-predicted three-dimensional structures of pea-derived antioxidant peptides. F1–F5 peptides, respectively. The color scale represents the pLDDT confidence score.

### 3.4 Molecular docking

Molecular docking was performed to investigate the predicted interactions between the five selected peptides (F1–F5) and Keap1. As shown in Fig. 4 and Table 2, all five peptides exhibited favorable predicted binding energies ranging from −10.1 to −8.5 kcal/mol. F1 showed the strongest predicted binding affinity with Keap1 (−10.1 kcal/mol), followed by F5 (−10.0 kcal/mol), F2 (−9.1 kcal/mol), F4 (−9.0 kcal/mol), and F3 (−8.5 kcal/mol).

**Fig. 4:**
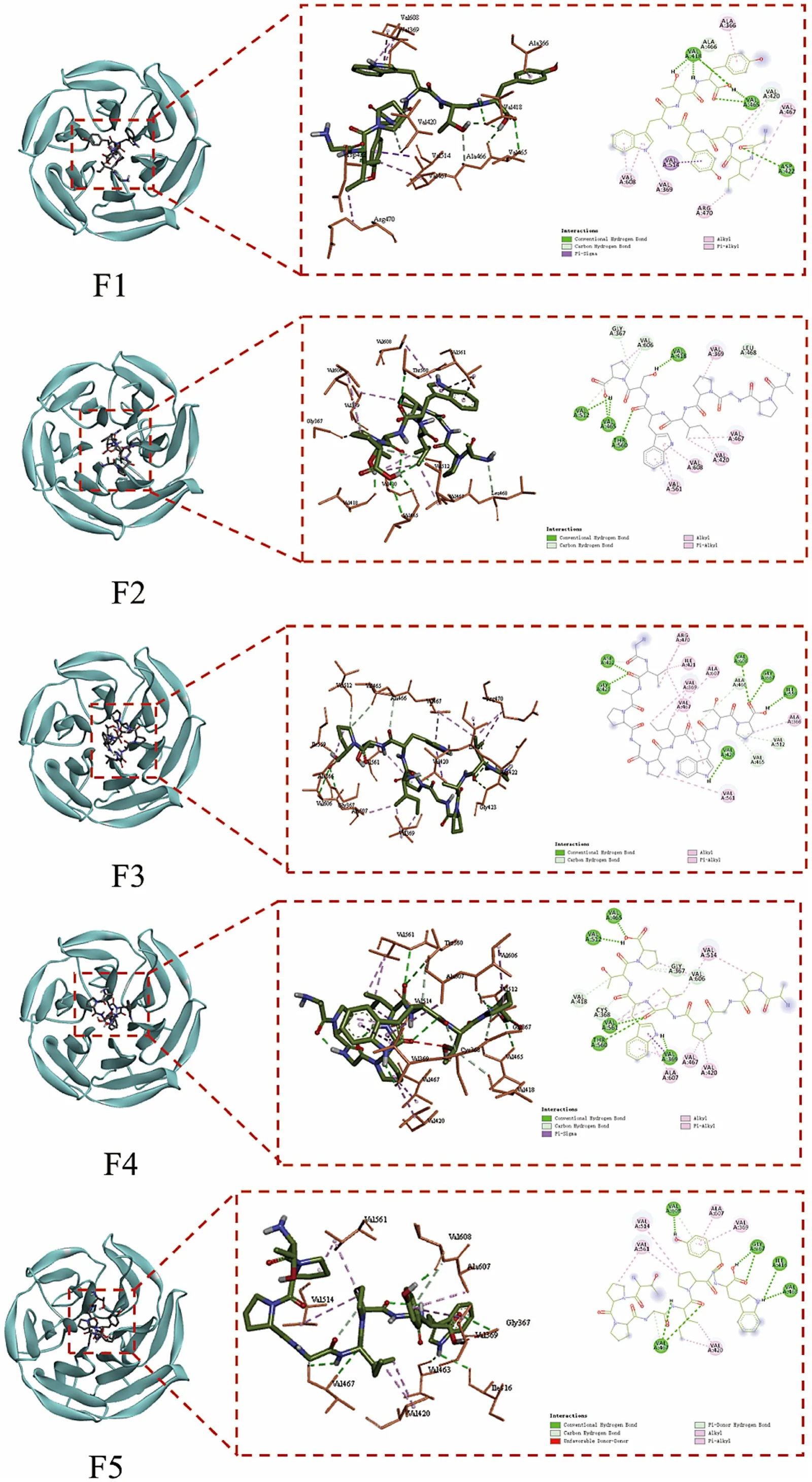
Predicted binding modes and molecular interactions between pea-derived antioxidant peptides and Keap1. (A–E) F1 (GIPYWTY), F2 (APGPIWTP), F3 (GVAPGPIWTP), F4 (APGPVWTP), and F5 (TPPGIPYW) complexes with Keap1, respectively. Three-dimensional and two-dimensional interaction patterns are shown for each peptide–Keap1 complex.

**Table 2:** Predicted binding energies and interaction residues of pea-derived antioxidant peptides with Keap1.

Peptide	Predicted binding energy (kcal/mol)	Number of hydrogen bonds	Keap1 residues involved in hydrogen bonding	Keap1 residues involved in hydrophobic interactions	Peptide residues involved in interactions
F1	−10.1	8	Val418, Val465, Val420, Asp422, Ala466	Ala366, Val467, Arg470, Val608, Val369, Val514	Gly, Thr, Tyr
F2	−9.1	9	Gly367, Val606, Val418, Leu468, Val512, Val456, Thr560	Val369, Val467, Val420, Val608, Val561	Trp, Thr, Pro
F3	−8.5	10	Asp422, Gly423, Val606, Ala466, Gly367, Ile559, Val512, Val465, Val420	Arg470, Ile421, Val369, Val467, Ala607, Ala366, Val561	Val, Trp, Pro
F4	−9.0	10	Val369, Thr560, Val561, Val418, Cys368, Val512, Val465, Gly367, Val606	Val514, Val467, Val420, Ala607	Val, Trp, Pro
F5	−10.0	9	Val608, Val463, Val467, Gly367, Ile416	Val561, Val514, Val369, Val420, Ala607	Ile, Tyr, Trp

The predicted peptide–Keap1 complexes were stabilized by multiple hydrogen bonds and hydrophobic interactions. Among the Keap1 residues involved in peptide recognition, Val420 and Val467 were consistently observed in the interaction interfaces of all five peptides. Other residues, including Val369, Val561, Gly367, Val418, Val512, and Val608, also frequently participated in the predicted interactions. These results indicate that the hydrophobic pocket of Keap1 may provide a favorable environment for peptide accommodation.

On the peptide side, aromatic and hydrophobic residues appeared to contribute substantially to Keap1 recognition. Trp and Pro were frequently involved in the predicted interaction regions, together with Tyr, Thr, Val, Gly, and Ile (Table 2). These residues may enhance peptide binding through hydrogen bonding, hydrophobic contacts, and aromatic interactions. Similar interaction patterns have been reported for food-derived antioxidant peptides, in which hydrogen bonding and hydrophobic interactions contributed to the stabilization of peptide–Keap1 complexes (Liu et al., 2024; Zhang, Yan, et al., 2024).

Among the five peptides, F1 exhibited the most favorable predicted binding energy, suggesting a potentially stronger interaction with Keap1. Therefore, F1 was selected for subsequent molecular dynamics simulation to further evaluate the stability and energetic characteristics of the peptide–protein complex. However, molecular docking provides only a computational prediction of binding modes and does not by itself demonstrate activation of the Keap1/Nrf2 signaling pathway.

### 3.5 Quantum chemical analysis

Frontier molecular orbital analysis was performed to characterize the electronic properties and potential reactivity of F1–F5. The HOMO represents the orbital most readily involved in electron donation, whereas the LUMO reflects the electron-accepting capability. The HOMO–LUMO energy gap (ΔE_HOMO–LUMO_) provides information on molecular electronic reactivity, with a smaller energy gap generally indicating higher electronic reactivity and easier electron redistribution (Igbokwe et al., 2024). As shown in Fig. 5 and Table 3, the E_HOMO_ values followed the order F2 > F4 > F3 > F5 > F1, whereas the E_LUMO_ values followed F1 < F5 < F3 < F2 < F4. The ΔE_HOMO–LUMO_ values increased in the order F1 (4.984 eV) < F5 (5.026 eV) < F2 (5.061 eV) < F4 (5.063 eV) < F3 (5.065 eV). Thus, F1 exhibited the smallest energy gap, suggesting relatively higher electronic reactivity among the five peptides.

**Fig. 5:**
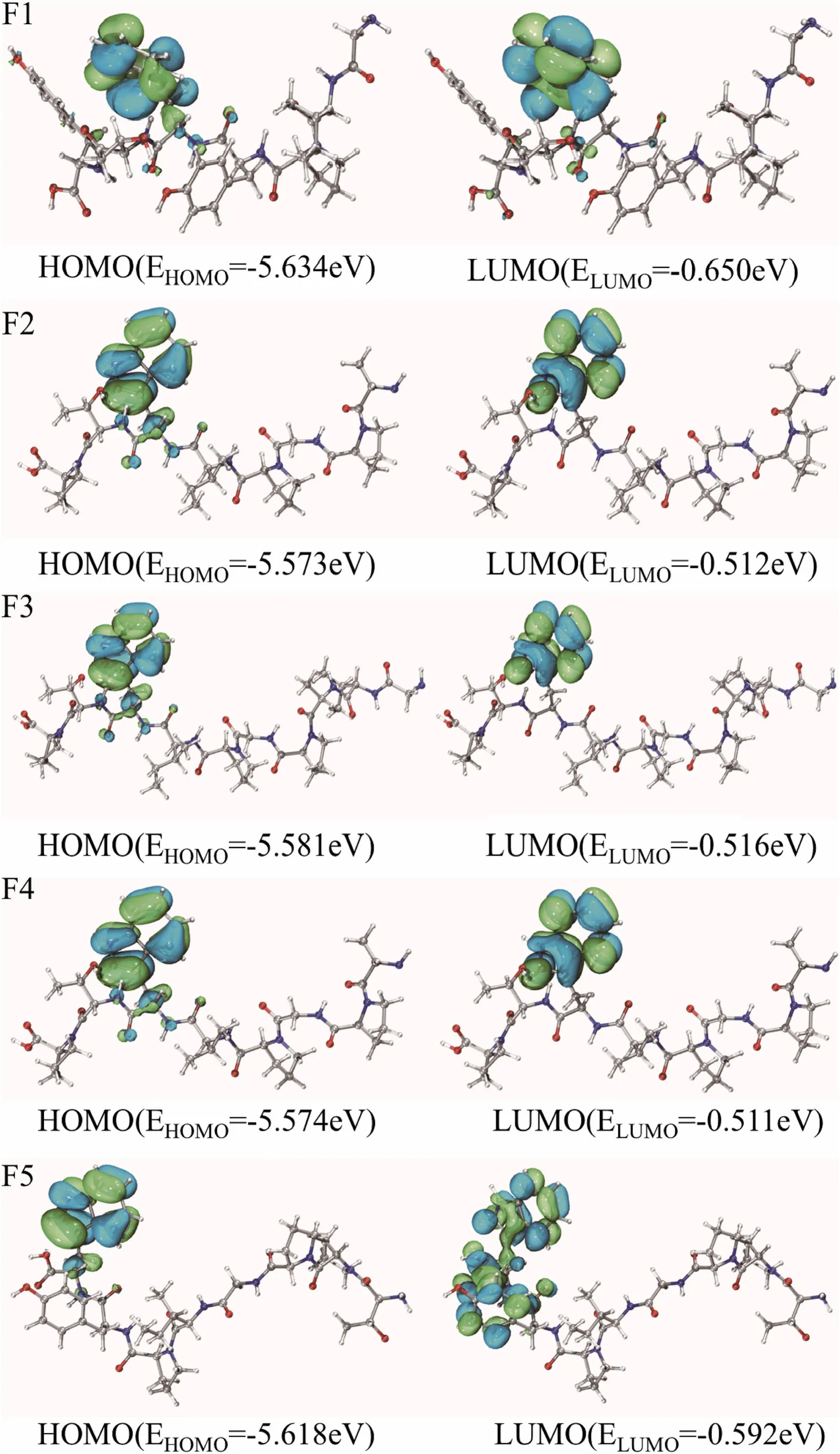
Frontier molecular orbital distributions of pea-derived antioxidant peptides. (A–E) Molecular structures, HOMO distributions, and LUMO distributions of F1 (GIPYWTY), F2 (APGPIWTP), F3 (GVAPGPIWTP), F4 (APGPVWTP), and F5 (TPPGIPYW), respectively. Red and blue surfaces represent different phases of the molecular orbitals. (For interpretation of the references to color in this figure legend, the reader is referred to the web version of this article.)

**Table 3:** Frontier molecular orbital energies and energy gaps of pea-derived antioxidant peptides (F1–F5).

Peptide sequence	E_HOMO_ (eV)	E_LUMO_ (eV)	ΔE_HOMO–LUMO_ (eV)
F1	−5.634	−0.650	4.984
F2	−5.573	−0.512	5.061
F3	−5.581	−0.516	5.065
F4	−5.574	−0.511	5.063
F5	−5.618	−0.592	5.026

***Note:*** EHOMO and ELUMO represent the energies of the highest occupied molecular orbital and lowest unoccupied molecular orbital, respectively. ΔEHOMO–LUMO represents the energy gap between HOMO and LUMO.

Fukui-function analysis was further used to identify atomic regions with higher predicted radical reactivity. The highest *f*^*0*^ values were observed at C82 of F1 (0.095), C79 of F2 (0.104), C102 of F3 (0.104), C76 of F4 (0.104), and C113 of F5 (0.073), while several neighboring carbon atoms also showed relatively high *f*^*0*^ values (Table S1). These results indicate that radical-reactive regions were heterogeneously distributed among different peptide structures. Such sequence-dependent electronic characteristics are consistent with previous quantum-chemical analyses of food-derived antioxidant peptides (Wang et al., 2025).

Mulliken charge analysis further revealed pronounced local charge polarization within F1–F5 (Table S2). Negative charges were mainly distributed on oxygen and nitrogen atoms, including O66 in F1 (−0.491), O69 in F2 (−0.476), O92 in F3 (−0.475), O66 in F4 (−0.476), and O87 in F5 (−0.448). In contrast, relatively positive charges were mainly associated with specific carbon atoms, including C74 in F1 (0.214), C77 in F2 (0.182), C100 in F3 (0.182), C74 in F4 (0.182), and C107 in F5 (0.428). Such charge polarization may contribute to electron redistribution during interactions with reactive species. Collectively, frontier orbital, Fukui-function, and Mulliken charge analyses provide complementary electronic-level insights into the potential antioxidant reactivity of F1–F5.

### 3.6 In vitro antioxidant activities of selected pea-derived antioxidant peptides

The in vitro radical-scavenging activities of the five synthetic peptides differed considerably among the three assays (Table 4). F2 exhibited the highest DPPH radical-scavenging activity (95.81 ± 0.27%) and hydroxyl radical-scavenging activity (50.20 ± 0.25%), both significantly higher than those of the other peptides (*P* < 0.05). In contrast, F5 showed the highest ABTS radical-scavenging activity (87.50 ± 0.24%), followed by F1 (78.93 ± 0.05%) (*P* < 0.05). F3 exhibited the lowest DPPH (43.19 ± 0.16%) and ABTS (21.39 ± 0.77%) radical-scavenging activities, whereas its hydroxyl radical-scavenging activity was not significantly different from those of F1, F4, and F5 (*P* > 0.05).

**Table 4:** In vitro antioxidant activities of selected pea-derived antioxidant peptides.

Peptide	DPPH radical-scavenging activity (%)	ABTS radical-scavenging activity (%)	Hydroxyl radical-scavenging activity (%)
GIPYWTY(F1)	52.20 ± 0.21	78.93 ± 0.05^b^	48.15 ± 0.44^b^
APGPIWTP(F2)	95.81 ± 0.27^a^	40.09 ± 0.42^c^	50.20 ± 0.25^a^
GVAPGPIWTP(F3)	43.19 ± 0.16^e^	21.39 ± 0.77^d^	47.33 ± 0.21^b^
APGPVWTP(F4)	49.48 ± 0.34^d^	40.97 ± 0.75^c^	47.50 ± 0.88^b^
TPPGIPYW(F5)	55.24 ± 0.24^b^	87.50 ± 0.24^a^	48.13 ± 0.77^b^

**Note:** Values are expressed as mean ± standard deviation (n = 3). Different lowercase superscript letters within the same column indicate significant differences among peptides (*P* < 0.05), as determined by one-way ANOVA followed by Tukey's multiple-comparison test.

The assay-dependent differences among F1–F5 indicate that antioxidant activity is influenced by peptide sequence, amino acid composition, and the accessibility of reactive residues rather than molecular weight alone. Aromatic and hydrophobic residues, such as Trp, Tyr, Phe, and Pro, may contribute to radical-scavenging reactions through hydrogen donation, electron transfer, and hydrophobic interactions (Matsui et al., 2018). Therefore, none of the peptides exhibited universally superior activity across all radical systems. Considering the integrated results from antioxidant assays, molecular docking, quantum-chemical calculations, and structural analyses, F1 was selected as the lead peptide for subsequent molecular investigation and walnut-oil stabilization studies.

### 3.7 Spectroscopic characterization and morphological analysis of pea-derived antioxidant peptides

UV–visible spectra revealed distinct differences between defatted pea protein and the five peptide samples (Fig. 6A). F1–F5 exhibited an absorption band at approximately 275–280 nm, which was associated with aromatic amino acid residues. Notably, all five peptides contained Trp, whereas F1 and F5 additionally contained Tyr, which may contribute to their characteristic UV absorption. Intrinsic fluorescence spectra showed higher fluorescence intensities for F1–F5 than for defatted pea protein under the measurement conditions, with emission maxima located approximately at 345–360 nm (Fig. 6B). These fluorescence characteristics were mainly attributed to Trp residues due to the conjugated π-electron system of the indole group (Liu et al., 2020).

**Fig. 6:**
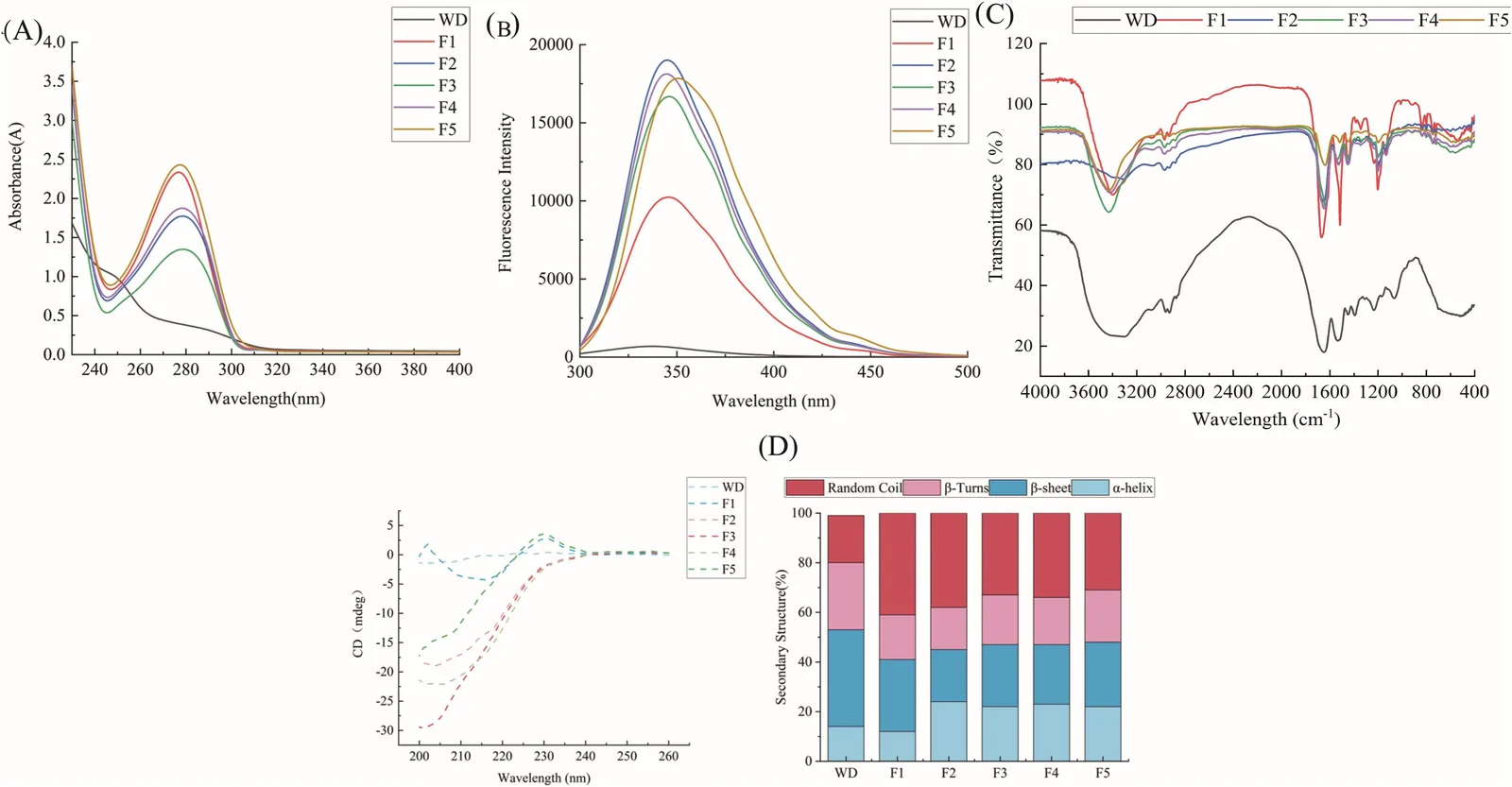
Spectroscopic characterization and secondary structure analysis of pea-derived antioxidant peptides. (A) UV–visible absorption spectra; (B) intrinsic fluorescence spectra; (C) Fourier-transform infrared (FTIR) spectra; and (D) circular dichroism (CD) spectra and BeStSel-predicted secondary structure compositions of defatted pea protein and F1–F5 peptides.

FTIR spectra exhibited characteristic peptide-related absorption bands in the amide I and amide II regions, together with a broad N–H/O–H stretching band (Fig. 6C). Differences in spectral profiles among F1–F5 reflected variations in their local chemical environments and conformational characteristics. Far-UV CD spectra further revealed distinct conformational features among the five peptides (Fig. 6D). BeStSel analysis indicated the presence of α-helices, β-sheets, β-turns, and other/unordered structural components. The proportion of the other/unordered component followed the order F1 > F2 > F4 > F3 > F5. Relatively flexible peptide conformations may improve the accessibility of reactive residues; however, secondary structure alone cannot fully explain differences in antioxidant activity (Xu et al., 2020). Collectively, these spectroscopic analyses demonstrate structural heterogeneity among F1–F5 and provide complementary information for understanding their different antioxidant behaviors.

Scanning electron microscopy further revealed distinct morphological characteristics among defatted pea protein and the five peptide samples (Fig. 7). Defatted pea protein (DP) exhibited a relatively compact and rough surface with localized pores, whereas F1–F5 displayed heterogeneous morphologies characterized by irregular fragments, lamellar features, and different aggregation patterns. F2 showed a comparatively loose and irregular aggregated structure, while F3 and F4 exhibited larger lamellar structures. In contrast, F5 displayed a relatively smooth surface with dispersed particulate aggregates. These morphological differences indicate distinct surface organization and aggregation behaviors among F1–F5. However, SEM observations alone cannot directly determine peptide secondary structures.

**Fig. 7:**
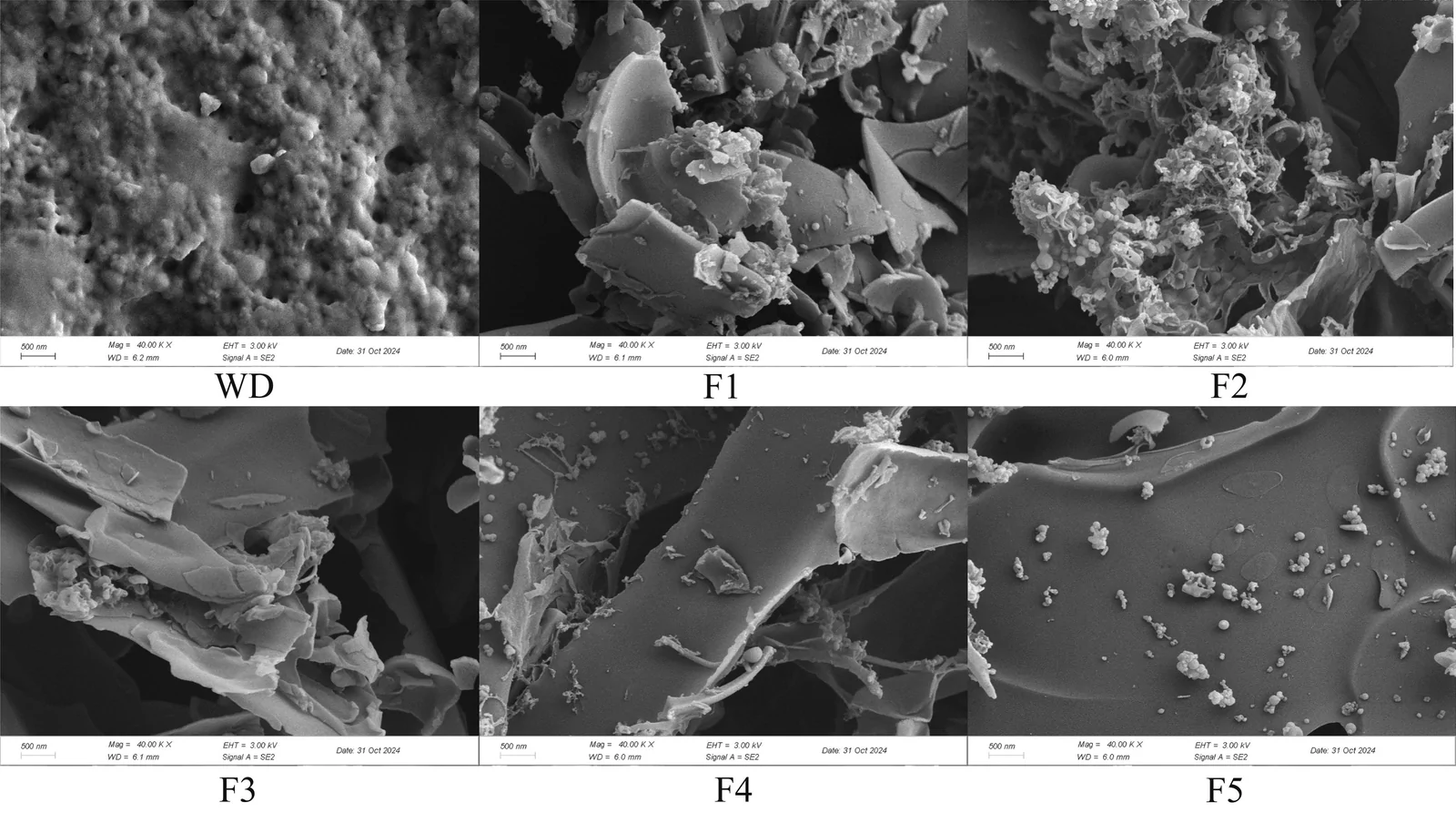
Scanning electron microscopy images of defatted pea protein and pea-derived antioxidant peptides (F1–F5).

### 3.8 Molecular dynamics simulation and stability analysis of the F1–Keap1 complex

Based on the integrated experimental and computational analyses, F1 (GIPYWTY) was selected as the lead peptide for further molecular dynamics investigation, and the F1–Keap1 complex was subjected to a 100 ns MD simulation to evaluate its dynamic stability. The number of intermolecular hydrogen bonds fluctuated throughout the trajectory, with approximately 5–7 hydrogen bonds observed during the initial stage, decreasing mainly to 2–4 during the intermediate period and increasing again toward the end of the simulation (Fig. 8A). These results indicate persistent but dynamically rearranging interactions between F1 and Keap1.

**Fig. 8:**
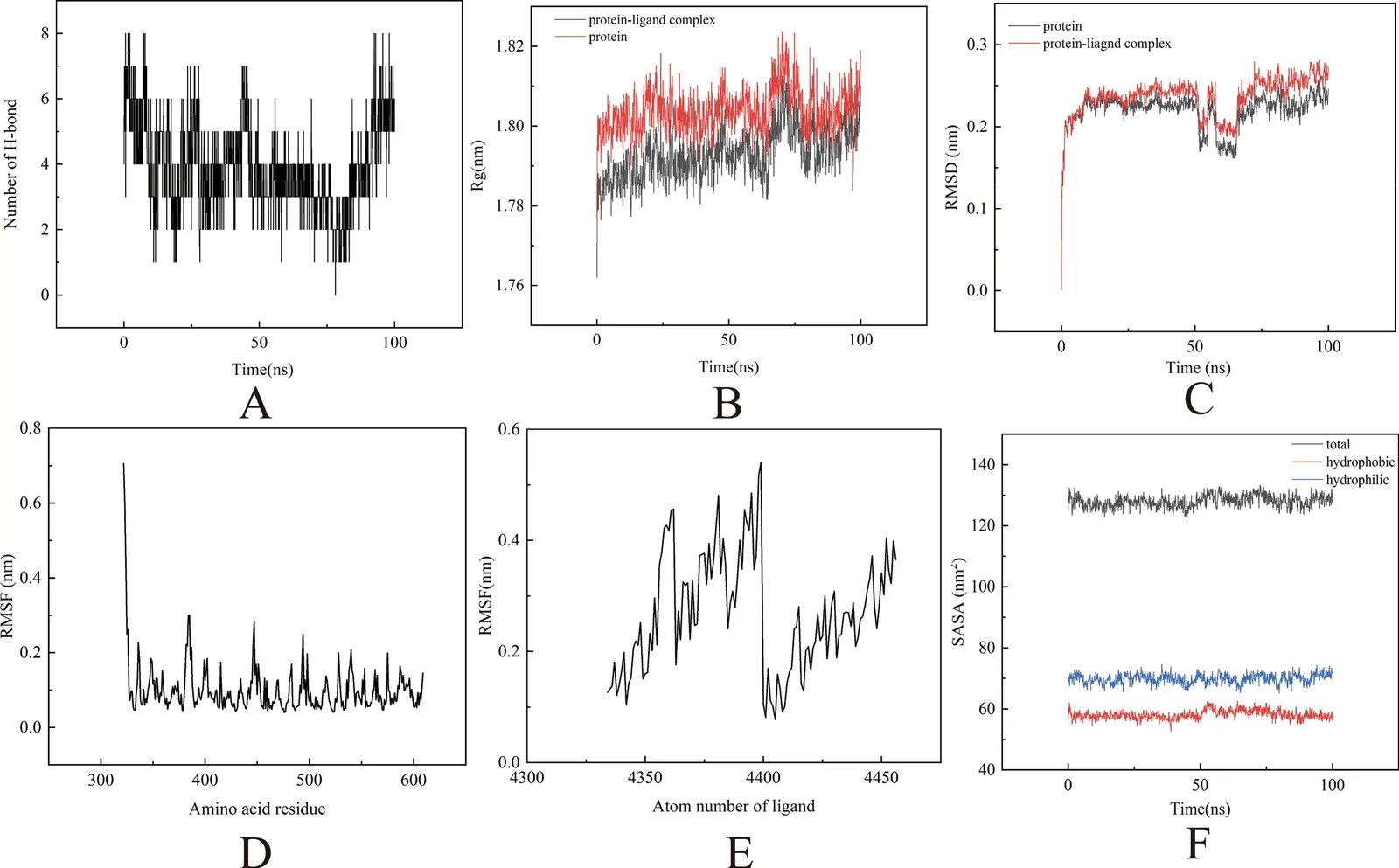
Molecular dynamics simulation analysis of the F1–Keap1 complex during a 100 ns simulation. (A) Number of intermolecular hydrogen bonds; (B) radius of gyration (Rg); (C) root mean square deviation (RMSD); (D) residue-based root mean square fluctuation (RMSF) of Keap1; (E) atom-based RMSF of F1; and (F) solvent-accessible surface area (SASA).

The Rg values of Keap1 and the F1–Keap1 complex remained within a narrow range of approximately 1.78–1.82 nm (Fig. 8B), indicating limited changes in global compactness. The total SASA remained relatively stable at approximately 125–130 nm^2^ throughout the simulation (Fig. 8F), suggesting no substantial changes in solvent exposure. RMSD values of Keap1 and the F1–Keap1 complex remained below approximately 0.30 nm after initial equilibration (Fig. 8C), with similar fluctuation patterns. Most Keap1 residues showed relatively limited fluctuations, generally within 0.05–0.20 nm, although several localized regions exhibited higher flexibility (Fig. 8D). In contrast, F1 atoms displayed greater fluctuations, reaching approximately 0.55 nm (Fig. 8E), indicating that the peptide retained internal flexibility within the binding region. Collectively, these parameters suggest that F1 binding did not induce substantial global destabilization of Keap1 during the simulation.

The free-energy landscape revealed a dominant low-energy basin centered at approximately RMSD = 0.22–0.24 nm and Rg = 1.79–1.81 nm, together with a smaller secondary basin around RMSD ≈ 0.17 nm and Rg ≈ 1.77–1.78 nm (Fig. 9A). These results indicate preferential sampling of energetically favorable conformational states. Secondary-structure analysis further showed that the overall structural pattern of Keap1 was largely maintained throughout the 100 ns trajectory, without extensive unfolding or structural remodeling (Fig. 9B).

**Fig. 9:**
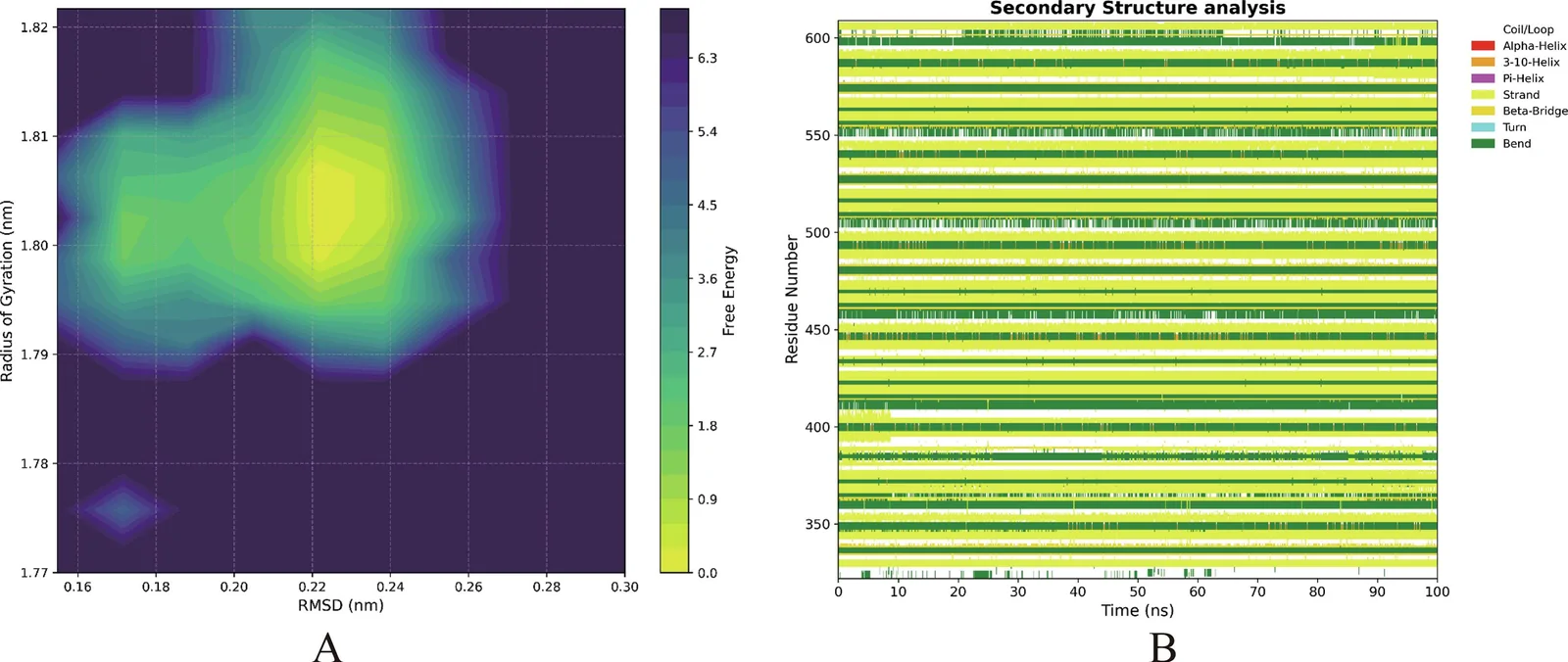
Conformational stability analysis of the F1–Keap1 complex during molecular dynamics simulation. (A) Free energy landscape based on RMSD and radius of gyration (Rg); and (B) secondary structure evolution of Keap1 during the 100 ns simulation.

Binding energetics were further evaluated using MM/PBSA analysis (Table 5). The calculated total binding free-energy estimate (ΔG_TOTAL_) was −36.73 kJ/mol. Van der Waals (−54.26 kJ/mol) and electrostatic interactions (−24.64 kJ/mol) contributed favorably to binding, whereas the polar solvation contribution (49.01 kJ/mol) opposed complex formation. The nonpolar solvation contribution (−6.83 kJ/mol) was favorable, and the favorable gas-phase interaction energy (−78.91 kJ/mol) partially compensated for the unfavorable solvation contribution (42.18 kJ/mol), resulting in an overall favorable binding free-energy estimate. Overall, MD and MM/PBSA analyses support a dynamically stable and energetically favorable interaction between F1 and Keap1. However, these computational results indicate binding potential and do not by themselves demonstrate activation of the Keap1/Nrf2 signaling pathway.

**Table 5:** MM/PBSA binding energy components of the F1–Keap1 complex.

Energy term	Average (kJ/mol)
ΔE_VDWAALS_	−54.26
ΔE_EL_	−24.64
ΔE_GB_	49.01
ΔE_SURF_	−6.83
ΔG_GAS_	−78.91
ΔG_SOLV_	42.18
ΔG_TOTAL_	−36.73

***Note:*** ΔE_VDWAALS_, van der Waals interaction contribution; ΔE_EL_, electrostatic interaction contribution; ΔE_GB_, polar solvation contribution; ΔE_SURF_, nonpolar solvation contribution; ΔG_GAS_, gas-phase interaction energy; ΔG_SOLV_, solvation free-energy contribution; and ΔG_TOTAL_, total MM/PBSA binding free-energy estimate.

### 3.9 Effect of F1 on the oxidative stability and antioxidant capacity of walnut oil

Walnut oil is rich in unsaturated fatty acids and is therefore susceptible to oxidative deterioration during processing and storage (Sun et al., 2024). Based on the integrated antioxidant assays and computational analyses, F1 was selected for further evaluation in a walnut oil model under accelerated oxidation conditions. As shown in Fig. 10, POV and AV increased progressively with oxidation time, whereas F1 supplementation significantly attenuated these increases compared with the control group, indicating delayed oxidative deterioration of walnut oil during the 10-day accelerated oxidation period.

**Fig. 10:**
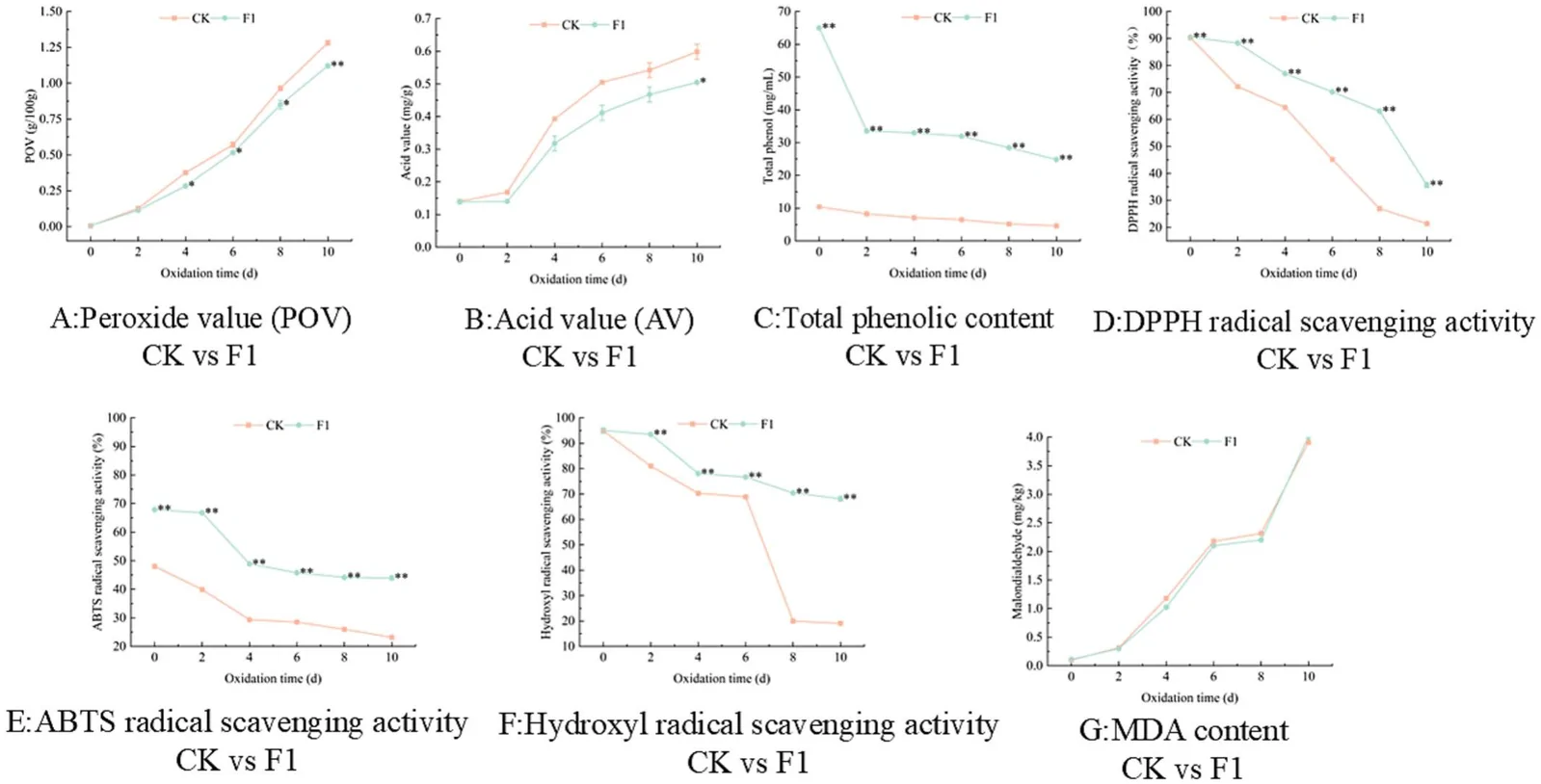
Effect of F1 peptide supplementation on oxidative stability and antioxidant capacity of walnut oil during accelerated oxidation at 60 °C. (A) Peroxide value (POV); (B) acid value (AV); (C) total phenolic content; (D) DPPH radical-scavenging activity; (E) ABTS radical-scavenging activity; (F) hydroxyl radical-scavenging activity; and (G) malondialdehyde (MDA) content.

The antioxidant capacity of walnut oil also declined progressively during oxidation, as reflected by decreases in DPPH, ABTS, and hydroxyl radical-scavenging activities. F1 supplementation partially preserved these antioxidant activities during accelerated oxidation. This protective effect may be associated with the radical-scavenging ability of F1 and the contribution of aromatic residues, particularly Trp and Tyr, which may participate in antioxidant reactions through hydrogen- and/or electron-transfer mechanisms. The contribution of aromatic residues to peptide antioxidant activity has also been reported previously (Matsui et al., 2018).

In contrast to POV and AV, MDA content did not differ significantly between the F1 and control groups during the 10-day oxidation period (*P* > 0.05). This difference may be attributed to the distinct formation kinetics and chemical characteristics of primary and secondary lipid oxidation products. POV mainly reflects the accumulation of lipid hydroperoxides during the early oxidation stage, whereas secondary oxidation products such as MDA are generated after hydroperoxide decomposition (Zhang et al., 2021). Therefore, inhibition of peroxide accumulation may not necessarily result in a significant reduction in MDA within the same oxidation period. Overall, these findings demonstrate that F1 retarded oxidative deterioration of walnut oil under accelerated conditions and highlight its potential as a natural antioxidant peptide for lipid-containing food systems.

## 4 Conclusion

Defatted pea protein hydrolysates were fractionated by ultrafiltration and Sephadex G-15 chromatography, followed by Nano LC–MS/MS identification and in silico screening of antioxidant peptide candidates. Five previously unreported peptides, GIPYWTY (F1), APGPIWTP (F2), GVAPGPIWTP (F3), APGPVWTP (F4), and TPPGIPYW (F5), were retained for experimental evaluation. Their radical-scavenging activities varied among DPPH, ABTS, and hydroxyl radical assays, demonstrating sequence- and assay-dependent antioxidant performance. Molecular docking suggested favorable interactions between F1–F5 and Keap1, while quantum-chemical analyses revealed distinct electronic properties and potential radical-reactive regions. Spectroscopic and morphological analyses further demonstrated structural heterogeneity among the five peptides.

Based on integrated experimental and computational evidence, F1 was selected as the lead peptide for further investigation. A 100 ns molecular dynamics simulation indicated that the F1–Keap1 complex maintained overall structural stability, and MM/PBSA analysis yielded a favorable binding free-energy estimate of −36.73 kJ/mol. In a walnut oil model, F1 supplementation attenuated increases in peroxide and acid values and partially preserved antioxidant capacity during 10 d of accelerated oxidation at 60 °C. Overall, these findings highlight GIPYWTY as a promising pea-derived antioxidant peptide candidate for delaying oxidative deterioration in lipid-containing food systems.

## CRediT authorship contribution statement

**Jingna Peng:** Writing – original draft, Software, Resources, Project administration, Methodology, Investigation, Data curation, Conceptualization. **Tingmei Qi:** Validation, Supervision, Investigation, Formal analysis. **Qi Lin:** Visualization, Supervision, Conceptualization. **Xinyong Zhang:** Validation, Supervision, Resources, Funding acquisition. **Yue Wang:** Visualization, Validation. **Yuanyuan Bao:** Writing – review & editing, Software, Resources, Methodology, Conceptualization.

## Funding declaration

This work was supported by the 10.13039/501100008871Yunnan Provincial Science and Technology Department, China (grant number **202401BD070001–048**). The funder had no role in the study design, data collection, analysis or interpretation, preparation of the manuscript, or the decision to submit the article for publication.

## Declaration of competing interest

The authors declare that they have no known competing financial interests or personal relationships that could have appeared to influence the work reported in this paper.

## Data Availability

Data will be made available on request.

## References

[bb0005] Abeynayake R., Zhang S., Yang W., Chen L. (2022). Development of antioxidant peptides from brewers’ spent grain proteins. LWT.

[bb0010] Abramson J., Adler J., Dunger J., Evans R., Green T., Pritzel A., Jumper J.M. (2024). Accurate structure prediction of biomolecular interactions with AlphaFold 3. Nature.

[bb0015] Fan X., Han Y., Sun Y., Zhang T., Tu M., Du L., Pan D. (2023). Preparation and characterization of duck liver-derived antioxidant peptides based on LC-MS/MS, molecular docking, and machine learning. LWT.

[bb0020] Igbokwe C.J., Feng Y., Louis H., Benjamin I., Quaisie J., Duan Y., Zhang H. (2024). Novel antioxidant peptides identified from coix seed by molecular docking, quantum chemical calculations and invitro study in HepG2 cells. Food Chemistry.

[bb0025] Jia J., Liu Q., Liu H., Yang C., Zhao Q., Xu Y., Wu W. (2024). Structure characterization and antioxidant activity of abalone visceral peptides-selenium in vitro. Food Chemistry.

[bb0030] Kim W., Zia M.B., Naik R.R., Ho K.K.H.Y., Selomulya C. (2025). Effects of polyphenols from Tasmannia lanceolata on structural, emulsifying, and antioxidant properties of pea protein. Food Chemistry.

[bb0035] Lee C.H., Hamdan N., Nyakuma B.B., Wong S.L., Wong K.Y., Tan H., Lee T.H. (2024). Purification, identification and molecular docking studies of antioxidant and anti-inflammatory peptides from edible bird’s Nest. Food Chemistry.

[bb0040] Li S., Wang Y., Zhou J., Wang J., Zhang M., Chen H. (2023). Structural characterization, cytotoxicity, and the antifungal mechanism of a novel peptide extracted from garlic (*Allium sativa* L.). Molecules.

[bb0045] Li W., Liu X., Liu Y., Zheng Z. (2025). High-accuracy identification and structure–activity analysis of antioxidant peptides via deep learning and quantum chemistry. Journal of Chemical Information and Modeling.

[bb0050] Lin H., Zhao J., Xie Y., Tang J., Wang Q., Zhao J., Xu M., Liu P. (2024). Identification and molecular mechanisms of novel antioxidant peptides from fermented broad bean paste: A combined in silico and in vitro study. Food Chemistry.

[bb0055] Liu C., Liu S., Zhang L., Wang X., Ma L. (2018). Partition behavior in aqueous two-phase system and antioxidant activity of flavonoids from *Ginkgo biloba*. Applied Sciences.

[bb0060] Liu W., Liu R., Qin Q., Wang H., Zhang X., Meng G. (2024). Molecular docking and molecular dynamics simulation of wheat gluten-derived antioxidant peptides acting through the Keap1–Nrf2 pathway. Journal of the Science of Food and Agriculture.

[bb0065] Liu Y., Xu J., Han L., Liu Q., Yang Y., Li Z., Liu Q. (2020). Theoretical research on excited states: Ultraviolet and fluorescence spectra of aromatic amino acids. Interdisciplinary Sciences: Computational LIfe Sciences.

[bb0070] Luo Q., Li X., Zhang Z., Chen A., Li S., Shen G., Hou X. (2022). Extraction of *Zanthoxylum* seed protein and identification of its simulated digestion products. LWT.

[bb0075] Matsui R., Honda R., Kanome M., Hagiwara A., Matsuda Y., Togitani T., Ikemoto N., Terashima M. (2018). Designing antioxidant peptides based on the antioxidant properties of the amino acid side-chains. Food Chemistry.

[bb0080] Micsonai A., Moussong É., Wien F., Boros E., Vadászi H., Murvai N., Kardos J. (2022). BeStSel: Webserver for secondary structure and fold prediction for protein CD spectroscopy. Nucleic Acids Research.

[bb0085] Ndiaye F., Vuong T., Duarte J., Aluko R.E., Matar C. (2012). Anti-oxidant, anti-inflammatory and immunomodulating properties of an enzymatic protein hydrolysate from yellow field pea seeds. European Journal of Nutrition.

[bb0090] Shanthakumar P., Klepacka J., Bains A., Chawla P., Dhull S.B., Najda A. (2022). The current situation of pea protein and its application in the food industry. Molecules.

[bb0095] Shi C., Liu M., Zhao H., Lv Z., Liang L., Zhang B. (2022). A novel insight into screening for antioxidant peptides from hazelnut protein: Based on the properties of amino acid residues. Antioxidants.

[bb0100] Sun L., Wang G., Xiong L., Yang Z., Ma Y., Qi Y., Li Y. (2024). Characterization of volatile organic compounds in walnut oil with various oxidation levels using olfactory analysis and HS-SPME-GC/MS. Current Research in Food Science.

[bb0105] Wang J., Kadyan S., Ukhanov V., Cheng J., Nagpal R., Cui L. (2022). Recent advances in the health benefits of pea protein (Pisum sativum): Bioactive peptides and the interaction with the gut microbiome. Current Opinion in Food Science.

[bb0110] Wang J., Liu J., John A., Jiang Y., Zhu H., Yang B., Wen L. (2022). Structure identification of walnut peptides and evaluation of cellular antioxidant activity. Food Chemistry.

[bb0115] Wang X., Bhullar K.S., Fu J., Chen B., Liu H., Su D., Zhou W. (2025). Unraveling novel antioxidant peptides from Asian swamp eel: Identification, in silico selection, and mechanistic insights through quantum chemical calculation and molecular docking. Food Chemistry.

[bb0120] Wei K., Wei Y., Zhou P., Zhu J., Peng L., Cheng L., Wei X. (2023). Preparation, characterization, and antioxidant properties of selenium-enriched tea peptides. Foods.

[bb0125] Wong F.-C., Xiao J., Wang S., Ee K.-Y., Chai T.-T. (2020). Advances on the antioxidant peptides from edible plant sources. Trends in Food Science & Technology.

[bb0130] Xu Y., Galanopoulos M., Sismour E., Ren S., Mersha Z., Lynch P., Almutaimi A. (2020). Effect of enzymatic hydrolysis using endo- and exo-proteases on secondary structure, functional, and antioxidant properties of chickpea protein hydrolysates. Journal of Food Measurement and Characterization.

[bb0135] Yin X., Yang Z., Shi W., Fan S., Guan X., Ren Y., Zhao H., Fan J., Wang M., Li J. (2025). Enrichment of antioxidant peptides by interfacial modification of oat polypeptides induced by zinc ions. International Journal of Biological Macromolecules.

[bb0140] Zhang J., Liu Q., Chen Q., Sun F., Liu H., Kong B. (2022). Synergistic modification of pea protein structure using high-intensity ultrasound and pH-shifting technology to improve solubility and emulsification. Ultrasonics Sonochemistry.

[bb0145] Zhang N., Li Y., Wen S., Sun Y., Chen J., Gao Y., Sagymbek A., Yu X. (2021). Analytical methods for determining the peroxide value of edible oils: A mini-review. Food Chemistry.

[bb0150] Zhang W., Hu S., Tang H., Zhong F., Lin Q., Li W., Deng J. (2026). Food-derived antioxidant peptides mediated multifunctional bioactivities and bioavailability-enhancing delivery strategies via the Nrf2/Keap1 signaling pathway. Food Chemistry.

[bb0155] Zhang Y., Liu Y., Li W., Han W. (2024). Antioxidant activity of soybean peptides and Keap1 protein: A combined in vitro and in silico analysis. LWT.

[bb0160] Zhang Y., Xie X., Yang Y., Huang L., Nan G., Wang X., Zhao H., Wu Q. (2025). Efficient production, computational screening, molecular docking, quantum chemical calculations, and application of novel antioxidant peptides from Tartary buckwheat in composite preservation films. Food Chemistry.

[bb0165] Zhao D., Liu X. (2023). Purification, identification and evaluation of antioxidant peptides from pea protein hydrolysates. Molecules.

[bb0170] Zhu Z., Xu Z., Li Y., Fan Y., Zhou Y., Song K., Meng L. (2024). Antioxidant function and application of plant-derived peptides. Antioxidants.

