# Supplementary material for: Discovery and molecular insights of a novel pea-derived antioxidant peptide: From quantum chemical insights to oxidative stability of walnut oil
Source: Food Chem X. 2026 Sep 18;39:104474. doi: 10.1016/j.fochx.2026.104474 (PMC13626792; doi:10.1016/j.fochx.2026.104474)
Supplement: Supplementary file 1 [file mmc1.docx]

**Table S1.** Complete Fukui function (*f^0^*) values of F1–F5

| F1 | | F2 | | F3 | | F4 | | F5 | |
| --- | --- | --- | --- | --- | --- | --- | --- | --- | --- |
| Atom | *f^0^* | Atom | *f^0^* | Atom | f^0^ | Atom | *f^0^* | Atom | *f^0^* |
| O66 | 0.012 | C67 | 0.010 | C90 | 0.010 | O66 | 0.012 | C86 | 0.013 |
| C67 | 0.015 | O69 | 0.012 | O92 | 0.011 | C67 | 0.014 | O87 | 0.017 |
| C70 | 0.075 | C70 | 0.014 | C93 | 0.014 | C70 | 0.076 | C91 | 0.010 |
| C71 | 0.088 | C73 | 0.076 | C96 | 0.076 | C71 | 0.096 | C92 | 0.022 |
| N72 | 0.055 | C74 | 0.096 | C97 | 0.096 | N72 | 0.056 | C94 | 0.018 |
| C74 | 0.033 | N75 | 0.056 | N98 | 0.056 | C74 | 0.036 | C96 | 0.010 |
| C75 | 0.035 | C77 | 0.036 | C100 | 0.036 | C75 | 0.040 | C97 | 0.024 |
| C76 | 0.091 | C78 | 0.040 | C101 | 0.040 | C76 | 0.104 | C99 | 0.021 |
| C78 | 0.058 | C79 | 0.104 | C102 | 0.104 | C78 | 0.061 | O101 | 0.010 |
| C80 | 0.079 | C81 | 0.061 | C104 | 0.061 | C80 | 0.086 | N105 | 0.010 |
| C82 | 0.095 | C83 | 0.086 | C106 | 0.086 | C82 | 0.101 | C106 | 0.012 |
| - | - | C85 | 0.100 | C108 | 0.101 | - | - | C107 | 0.0342 |
| - | - | - | - | - | - | - | - | O108 | 0.0380 |
| - | - | - | - | - | - | - | - | C109 | 0.017 |
| - | - | - | - | - | - | - | - | C112 | 0.066 |
| - | - | - | - | - | - | - | - | C113 | 0.073 |
| - | - | - | - | - | - | - | - | N114 | 0.050 |
| - | - | - | - | - | - | - | - | C116 | 0.023 |
| - | - | - | - | - | - | - | - | C117 | 0.023 |
| - | - | - | - | - | - | - | - | C118 | 0.063 |
| - | - | - | - | - | - | - | - | C120 | 0.040 |
| - | - | - | - | - | - | - | - | C122 | 0.053 |
| - | - | - | - | - | - | - | - | C124 | 0.061 |
| - | - | - | - | - | - | - | - | O129 | 0.022 |

***Note:*** *f^0^ represents the Fukui function for radical attack. Higher f^0^ values indicate greater local susceptibility to radical reactions. The atom with the highest f^0^ value in each peptide represents a major predicted radical-reactive site.*

**Table S2.** Mulliken atomic charges of selected potential reactive sites in F1–F5

| F1 | | F2 | | F3 | | F4 | | F5 | |
| --- | --- | --- | --- | --- | --- | --- | --- | --- | --- |
| Atom | Charge | Atom | Charge | Atom | Charge | Atom | Charge | Atom | Charge |
| O66 | -0.490502 | C67 | -0.078458 | C90 | -0.076995 | O66 | -0.476119 | C86 | 0.385128 |
| C67 | -0.076101 | O69 | -0.476470 | O92 | -0.475400 | C67 | -0.143583 | O87 | -0.448055 |
| C70 | -0.194855 | C70 | -0.141501 | C93 | -0.144748 | C70 | -0.230226 | C91 | -0.153125 |
| C71 | 0.066980 | C73 | -0.229682 | C96 | -0.231937 | C71 | 0.100560 | C92 | -0.089327 |
| N72 | -0.424271 | C74 | 0.101568 | C97 | 0.100855 | N72 | -0.430373 | C94 | -0.128646 |
| C74 | 0.213527 | N75 | -0.430147 | N98 | -0.430765 | C74 | 0.182180 | C96 | 0.145920 |
| C75 | -0.134543 | C77 | 0.182108 | C100 | 0.181944 | C75 | -0.097333 | C97 | -0.129152 |
| C76 | -0.179443 | C78 | -0.098757 | C101 | -0.096180 | C76 | -0.101356 | C99 | -0.095151 |
| C78 | -0.125873 | C79 | -0.101286 | C102 | -0.101325 | C78 | -0.154115 | O101 | -0.423293 |
| C80 | -0.142275 | C81 | -0.153977 | C104 | -0.154577 | C80 | -0.146360 | N105 | -0.373364 |
| C82 | -0.087594 | C83 | -0.146667 | C106 | -0.146258 | C82 | -0.086865 | C106 | -0.079983 |
| - | - | C85 | -0.086633 | C108 | -0.086588 | - | - | C107 | 0.427860 |
| - | - | - | - | - | - | - | - | O108 | -0.409246 |
| - | - | - | - | - | - | - | - | C109 | -0.151666 |
| - | - | - | - | - | - | - | - | C112 | -0.193957 |
| - | - | - | - | - | - | - | - | C113 | 0.094197 |
| - | - | - | - | - | - | - | - | N114 | -0.436978 |
| - | - | - | - | - | - | - | - | C116 | 0.193666 |
| - | - | - | - | - | - | - | - | C117 | -0.112281 |
| - | - | - | - | - | - | - | - | C118 | -0.106849 |
| - | - | - | - | - | - | - | - | C120 | -0.159530 |
| - | - | - | - | - | - | - | - | C122 | -0.144891 |
| - | - | - | - | - | - | - | - | C124 | -0.085941 |
| - | - | - | - | - | - | - | - | O129 | -0.336484 |

***Note:*** *Positive and negative values represent electron-deficient and electron-rich atomic regions, respectively.*
